# Caspases and programmed cell death in sepsis: mechanisms, pathophysiology, and therapeutic targets

**DOI:** 10.3389/fimmu.2026.1879033

**Published:** 2026-07-17

**Authors:** Haibei He, Xiuli Xie, Lisui Zhou, Yumeng Lin, Aijun Liu, Peng Guo, Yu Zhu, Yu Tang, Zhongyu Han, Yi Wang, Haoran Chen

**Affiliations:** 1 Department of Intensive Care Medicine, Chengdu Xinhua Hospital Affiliated to North Sichuan Medical College, Chengdu, China; 2 School of Pharmacy, Xiamen University, Xiamen, China; 3 Nanjing Tongren Hospital, School of Medicine, Southeast University, Nanjing, China; 4 Department of Respiratory Medicine, Chengdu Xinhua Hospital Affiliated to North Sichuan Medical College, Chengdu, China; 5 Zhongda Hospital, Southeast University, Nanjing, China

**Keywords:** apoptosis, caspase, necroptosis, PANoptosis, pyroptosis, sepsis

## Abstract

Sepsis represents a critical medical condition characterized by organ impairment resulting from an uncontrolled systemic reaction to pathogenic infection, posing substantial difficulties in intensive care management. As a key pathological mechanism, programmed cell death (PCD) critically contributes to sepsis development, and caspases act as the central molecular hub governing multiple PCD modalities, including apoptosis, necroptosis, pyroptosis, and PANoptosis. In this manuscript, we review the classification and structural features of caspases, illustrate their molecular mechanisms in regulating the four PCD pathways, and clarify the dual pathological roles of these PCD forms in sepsis-induced immune dysregulation and organ damage. We also summarize therapeutic strategies targeting caspases and provide a theoretical basis for developing novel targeted treatments for sepsis.

## 1 Introduction

Sepsis represents a critical medical condition characterized by organ impairment resulting from an uncontrolled systemic reaction to pathogenic infection, posing substantial difficulties in intensive care management (1, 2). Globally, it has been identified as a public health concern that endangers human life and well-being. Epidemiological data indicate that the global burden of sepsis-related mortality declined from 16.5 million (representing 35.8% of all global deaths) in 1990 to 14 million (24.8%) in 2017 (3). However, the outbreak and global pandemic of COVID-19 reversed this downward trend: in 2021, sepsis-associated fatalities surged to 21.4 million cases, representing 32.8% of global deaths, with nearly 8 million of these deaths directly linked to COVID-19 infection (4). The currently widely accepted third international consensus definitions for sepsis and septic shock (Sepsis-3) clearly emphasizes that the core essence of sepsis lies in infection-induced dysregulation of host immune-inflammatory responses, rather than the infection itself or systemic inflammatory response syndrome (SIRS) (2, 5, 6). This immune-inflammatory dysregulation can further induce multiple organ dysfunction syndrome (MODS) and even progress to septic shock in severe cases, leading to extremely poor prognosis. Common severe complications of sepsis include sepsis-associated acute lung injury (SA-ALI), sepsis-associated acute kidney injury (SA-AKI), sepsis-associated encephalopathy (SAE), and sepsis-induced myocardial injury (SIMI) (7–9). Despite advances in medical technology that have bolstered the early identification and standardized management of sepsis, current sepsis care remains anchored in basic interventions, such as fluid resuscitation, anti-infection intervention, organ function maintenance, anti-inflammatory modulation, and nutritional supplementation (10). However, the clinical characteristics of sepsis, including acute onset, rapid progression and high severity, coupled with the impacts of the widespread prevalence of multidrug-resistant bacteria, adverse effects of antimicrobial agents and various treatment-related adverse events, further exacerbate the complexity of sepsis treatment, which not only prolongs the disease course but also results in suboptimal patient prognosis (11, 12). Therefore, exploring novel therapeutic strategies and identifying potential molecular targets holds profound clinical and translational significance.

**Figure 1:**
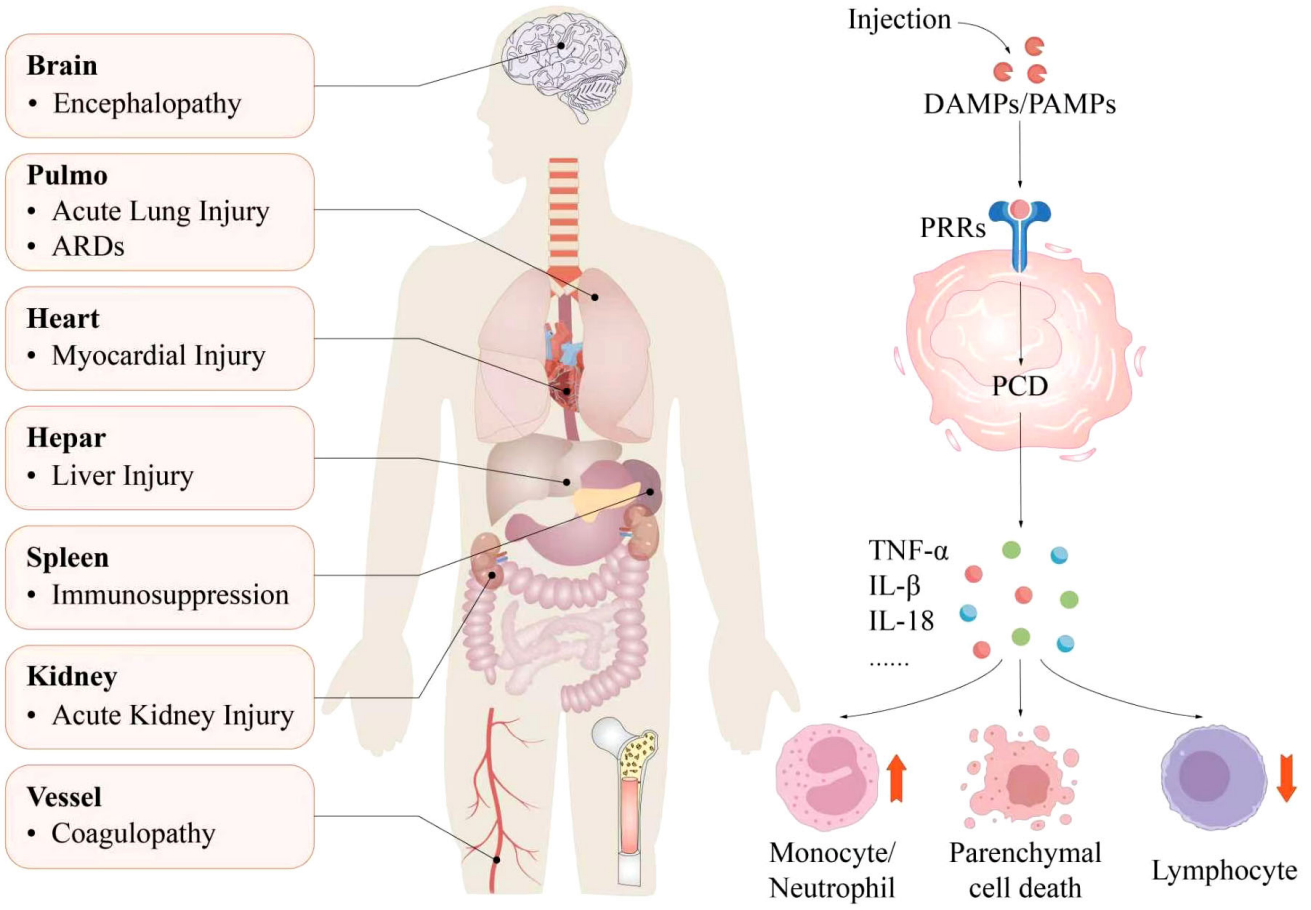
Overview of programmed cell death (PCD)-driven multi-organ dysfunction syndrome (MODS) in sepsis. Sepsis is triggered by pathogen-associated molecular patterns (PAMPs) and damage-associated molecular patterns (DAMPs) released during infection or tissue injury, which are recognized by pattern recognition receptors (PRRs) on immune and parenchymal cells. This recognition activates various forms of programmed cell death (PCD), leading to three key cellular outcomes: enhanced activation of monocytes/neutrophils, parenchymal cell death, and lymphocyte depletion. These cellular responses collectively drive systemic inflammation and multi-organ dysfunction, manifesting as encephalopathy (brain), acute lung injury/ARDS (lung), myocardial injury (heart), liver injury (liver), immunosuppression (spleen), acute kidney injury (kidney), and coagulopathy (vessels).

Programmed cell death (PCD), a self-regulated pattern of cellular demise involving multiple modalities, serves not only as a core mediator linking infectious signal transduction to tissue and organ damage in sepsis, but also occupies an irreplaceable critical position throughout the entire course of sepsis onset, progression, and prognosis. During early sepsis, the host-initiated immune defense responses to induce PCD in immune cells; at this stage, PCD exerts anti-infective effects by participating in innate immune responses, inhibiting intracellular pathogen replication, and activating the phagocytic and killing functions of immune cells. However, once PCD processes become dysregulated, they will excessively trigger inflammatory cascades in adjacent cells and tissues, subsequently deepening inflammatory injury. Throughout the pathological progression of sepsis, multiple PCD pathways become abnormally activated, triggering a cascade of events that encompass cytokine storm, immune-thrombotic dysregulation, and endothelial injury. Such pathological changes subsequently mediate capillary leakage and distributive shock, which in turn contribute to the onset of irreversible critical complications such as SA-AKI, SAMI, and SAE (**Figure 1**) (13, 14). Given the central regulatory role of PCD in sepsis pathophysiology, understanding how its different forms work is genuinely important. Cysteinyl-aspartate-specific proteases (caspases), an evolutionarily conserved family of cysteine proteases, serve as a central molecular hub governing multiple PCD modalities, including apoptosis, necroptosis, pyroptosis, and PANoptosis (15). Different caspase family members recognize distinct substrates and are activated in a tightly regulated sequence. In sepsis, this mechanism determines the survival or demise of infected and injured cells. Therefore, figuring out how caspases are regulated and wired into these networks is an important step toward understanding what goes wrong in sepsis and, ultimately, designing therapies that hit the right targets.

In this manuscript, we comprehensively review the molecular mechanisms underlying the activation of caspase-related PCD modalities (apoptosis, necroptosis, pyroptosis, and PANoptosis) during sepsis progression, clarify their regulatory characteristics and pathological roles in sepsis-induced immune disorders and organ damage, and further explore the potential translational value of targeting caspases or related PCD pathways for sepsis therapy.

## 2 Classification of caspases

**Figure 2:**
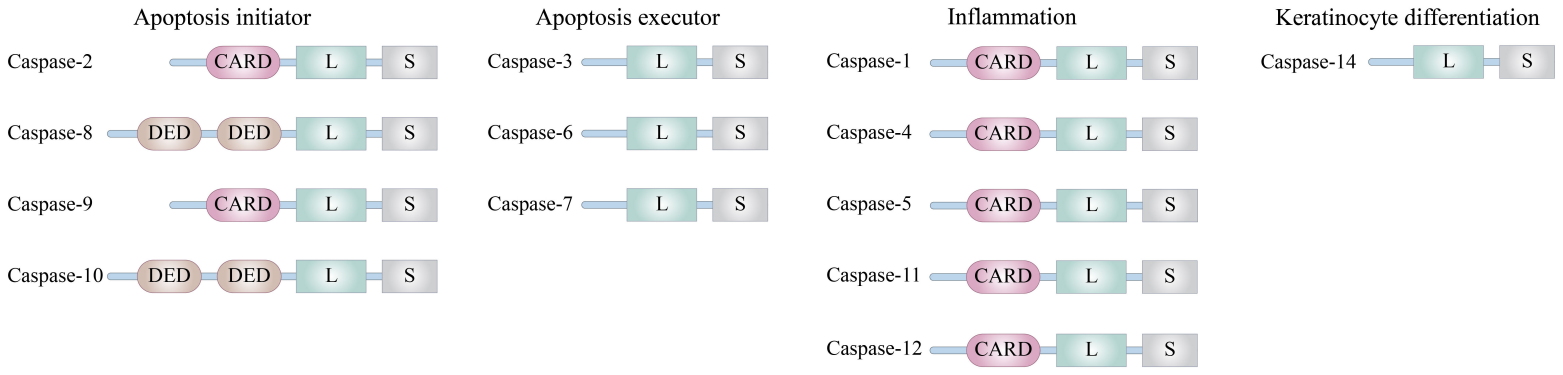
Functional classification and domain structure of caspases. Caspases are categorized by function: apoptosis initiators, apoptosis executors, inflammatory caspases, and Caspase-14 (involved in keratinocyte differentiation). Initiator/inflammatory caspases contain N-terminal recruitment domains (CARD or DED), while all caspases possess conserved large (L) and small (S) catalytic subunits.

Caspases constitute to an evolutionarily conserved family of cysteine-dependent proteolytic enzymes, among which 14 members (caspase-1 to caspase-14) have been identified to date (16). Functionally, these proteases are divided into three subgroups: apoptosis initiator caspases (caspase-2, -8, -9 and -10), apoptosis executor caspases (caspase-3, -6 and -7), and inflammatory caspases (caspase-1, -4, -5, -11 and -12) (16). Caspase-13 is not expressed in humans (17). Caspase-14 is involved in keratinocyte differentiation and does not belong to the category of canonical caspases (**Figure 2**) (18).

Caspases are universally expressed across different cell types and exist as inactive zymogens (pro-caspases) under physiological conditions (19). Structurally, they have a C-terminal effector domain made up of two subunits. The large subunit contains a conserved cysteine active site, serving as the core functional element for mediating substrate peptide bond cleavage (20). Some caspases also harbor a non-enzymatic N-terminal domain, including the death effector domain (DED) and the caspase activation and recruitment domain (CARD) (21). DED and CARD both belong to the death domain (DD) superfamily that regulate PCD and inflammatory signaling. They activate caspase cascades through selective homotypic and heterotypic interactions that build signaling platforms (21, 22). Effector caspases lack these domains entirely and depend on cleavage by initiator caspases to gain activity (23). This structural split lets caspases control multiple PCD pathways while keeping the decision process tightly regulated (24). Since PCD dysregulation is central to sepsis, understanding how caspases drive these processes is essential. Here, we examine how apoptosis, necroptosis, pyroptosis, and PANoptosis are executed, focusing on what distinguishes these pathways and how they drives immune suppression and organ injury in sepsis.

## 3 Caspases in apoptosis

Apoptosis is a typical highly conserved PCD, characterized by distinct morphological and biochemical features (25). Morphologically, apoptotic cells exhibit characteristic alterations such as shrinkage, chromatin compaction, nuclear breakdown, and apoptotic body formation (26). In contrast to other types of PCD, apoptotic cells are rapidly phagocytosed by adjacent phagocytes without triggering inflammation. Biochemically, apoptosis is driven by a cascade reaction of caspases (27). Apoptosis activators are triggered by specific death signals, then cleave and activate apoptosis executioners, which in turn degrade key cellular substrates (such as poly (ADP-ribose) polymerase (PARP) and cytoskeletal proteins) to execute the apoptotic program (28). Apoptosis is divided into the intrinsic (mitochondrial) pathway and the extrinsic (death receptor, DR) pathway based on the source of death signals, with caspase-9 and caspase-8 serving as their respective initiators. Intracellular stressors like DNA damage and oxidative stress trigger the intrinsic apoptotic pathway. Upregulation of pro-apoptotic proteins and downregulation of anti-apoptotic proteins lead to increased mitochondrial membrane permeability (29). This facilitates cytochrome c leakage from the mitochondrial intermembrane space to the cytoplasm, subsequently initiating caspase-9 and its downstream effector caspase cascade (30). Conversely, engagement of cell-surface DRs by extracellular death ligands triggers the extrinsic pathway. Ligand binding (such as Fas-FasL) causes the receptor’s cytoplasmic DD to recruit adaptor proteins including Fas-associated death domain (FADD). FADD then use its DED motifs to assemble procaspase-8 into the death-inducing signaling complex (DISC) (31). Pro-caspase-8 is activated via proximity-induced autocleavage within DISC, and active caspase-8 then transmits the apoptotic signal by activating downstream effector caspases, executing the apoptotic program (32).

## 4 Caspases in necroptosis

Necroptosis is a mixed lineage kinase domain-like protein (MLKL)-dependent PCD form. Unlike apoptosis, necroptosis forms polymeric MLKL membrane pores, causing cell rupture and intracellular content release, which triggers robust inflammatory responses (33).

Caspase-8 is the main protease in necroptosis regulation. Necroptosis and extrinsic apoptosis share the same DR-mediated initiating signals. Whether cells undergo necroptosis or apoptosis is largely determined by the activation level of caspase-8 (34). Taking the TNFR1-mediated pathway as an example: when TNF-α binds to cell-surface TNFR1, the receptor undergoes conformational changes and recruits TNFR-associated death domain protein (TRADD), receptor-interacting serine/threonine-protein kinase 1 (RIPK1), cellular inhibitor of apoptosis protein 1/2 (cIAP1/2), and other adaptor molecules to form the TNFR1-initiated signaling complex (35, 36). This complex exhibits distinct bidirectional functional tendencies, and the subsequent mode of PCD it mediates is determined by caspase-8. When caspase-8 is suppressed, RIPK1 binds to RIPK3 and mediate its phosphorylation (37). Phosphorylated RIPK3 further recruits and phosphorylates MLKL; phosphorylated MLKL then undergoes oligomerization, translocates to the cell membrane, and inserts into the lipid bilayer, forming non-ion-selective pores (38). This process induces massive Ca^2+^ influx, cell swelling, and rupture, ultimately cause leakage of damage-associated molecular patterns (DAMPs), initiating local inflammatory responses (39). So, necroptosis can serve as an alternative PCD pathway when the apoptotic pathway is inhibited. When caspase-8 is active, RIPK1 and RIPK3 are specifically cleaved, abrogating their ability to mediate necroptosis (40). Meanwhile, caspase-8 activates caspase-3, switching the cell from necroptosis to apoptosis. Under physiological conditions, caspase-8 forms a heterodimer with cellular-FLICE inhibitory protein (cFLIP). CFLIP is a caspase-8 homolog devoid of proteolytic activity (41). The caspase-8-cFLIP heterodimer exerts scaffolding functions by binding RIPK1 to block the assembly of RIPK1-RIPK3 necrosomes (42). Meanwhile, this heterodimer fails to activate caspase-3, thereby inhibiting both apoptosis and necroptosis and sustaining cell survival (43).

## 5 Caspases in pyroptosis

Pyroptosis is a gasdermin (GSDM)-dependent PCD. It is characterized by the oligomerization of the GSDM N-terminal pore-forming domain (N-PFD), which assembles pores on the plasma membrane. This process causes cell rupture, intracellular contents release, and inflammatory responses (44). GSDM family members GSDMA–GSDME are centrally involved in the core processes of pyroptosis. All of these proteins have two conserved structural domains: the N-PFD and the C-terminal repressive domain (C-RD) (45). Physiologically, C-RD inhibits N-PFD cytotoxicity via direct interaction. When pyroptotic signaling is activated, the interdomain linker region is specifically cleaved, enabling N-PFD release from C-RD repression. Free N-PFD then translocates to the cytoplasmic membrane, oligomerizes to form lipid bilayer pores, and drives interleukin-1β (IL-1β) and IL-18 secretion, thereby mediating pyroptosis (46).

Caspases act as the major GSDM-cleaving proteases, apart from granzyme and neutrophil elastase (47, 48). Caspase-mediated pyroptosis can be categorized into two distinct subtypes: inflammatory caspase-mediated pyroptosis and apoptosis-associated caspase-mediated pyroptosis (49). Specifically, the former comprises the canonical pathway and the non-canonical pathway, which primarily activate GSDMD (50). In the canonical pathway, caspase-1 activation depends on inflammasome assembly. Inflammasomes are composed of pattern recognition receptors (PRRs), apoptosis-associated speck-like protein containing a CARD (ASC), and pro-caspase-1 (51). PRRs harbor specialized domains for recognizing pathogen-associated molecular patterns (PAMPs) and DAMPs, enabling them to bind specifically to abnormal intracellular signals. They also carry pyrin domain (PYD) or CARD motifs for recruiting ASC or pro-caspase-1, triggering complex assembly (52). ASC bridges PRRs and pro-caspase-1 via its own PYD and CARD domains (53). After inflammasome assembly, caspase-1 activates, cleaves GSDMD, and promotes IL-1β and IL-18 maturation and release (54).

Beyond the canonical pathway, lipopolysaccharide (LPS) can trigger inflammasome-independent pyroptosis through direct binding to caspase-4/5 in humans or caspase-11 in mice, ultimately activating GSDMD (55). The ensuing K^+^ efflux acts as a secondary signal that activates the NOD-like receptor family pyrin domain containing 3 (NLRP3) inflammasome, bridging the non-canonical and canonical routes to ramp up inflammatory output (56).

**Figure 3:**
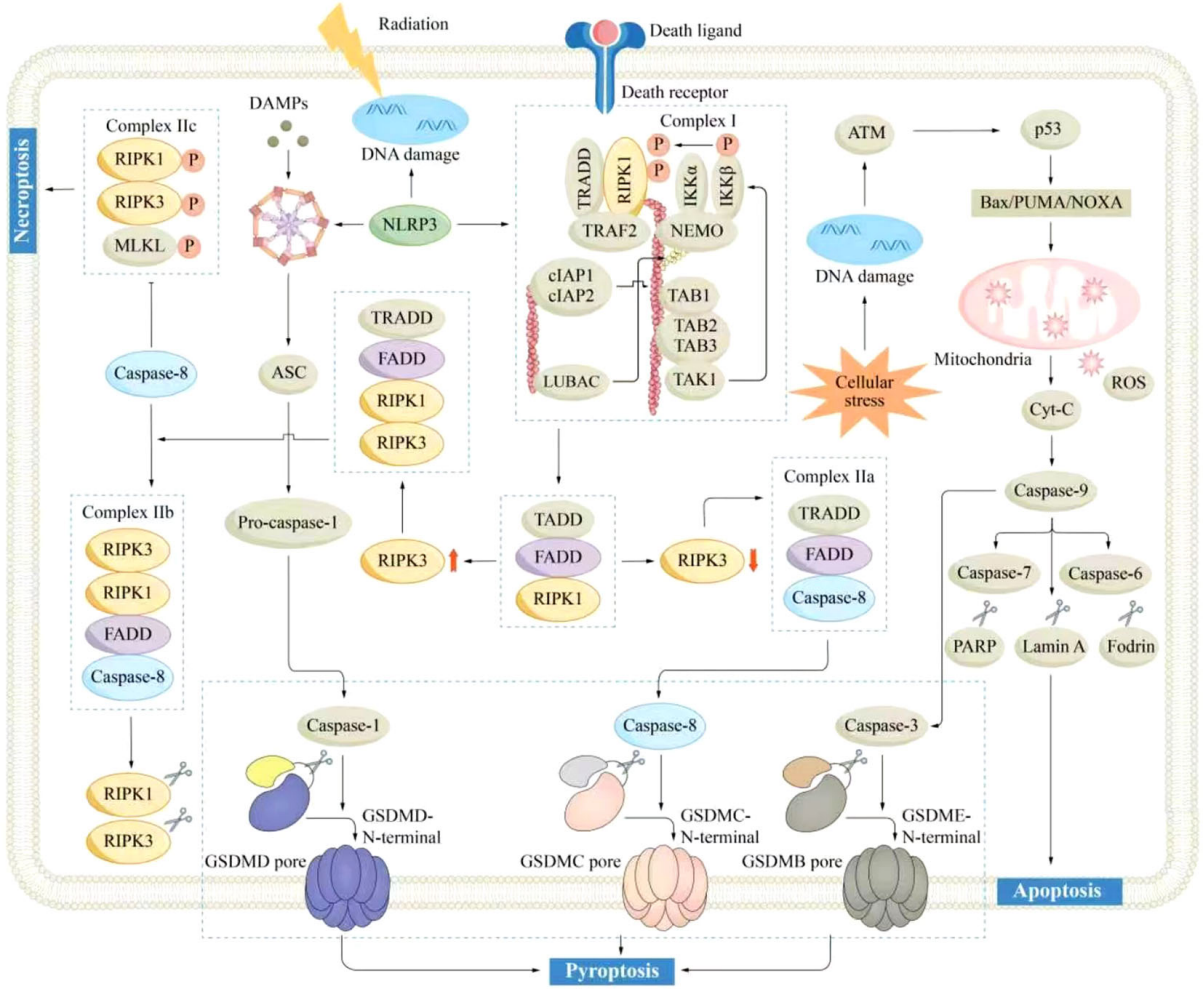
Molecular signaling pathways of apoptosis, necroptosis, and pyroptosis. Necroptosis is initiated when death ligands bind death receptors, forming Complex I, which normally promotes cell survival via NF-κB activation. Under conditions of cIAP depletion or RIPK1 inhibition, Complex I transitions to Complex IIa, driving apoptosis, or to Complex IIb and Complex IIc, leading to necroptosis via MLKL phosphorylation. Caspase-8 cleaves RIPK1 and RIPK3 to suppress necroptosis. Apoptosis can also be triggered by intrinsic stimuli (radiation, cellular stress, DNA damage), activating ATM-p53-Bax/PUMA/NOXA signaling to induce mitochondrial outer membrane permeabilization, cytochrome c release, and caspase-9 activation, which subsequently cleaves caspase-3, -6, and -7 to execute apoptosis. Pyroptosis is activated by DAMPs or DNA damage-induced NLRP3 inflammasome assembly with ASC and Pro-caspase-1, leading to Caspase-1 activation and GSDMD cleavage. Caspase-1 also activates Caspase-8, which cleaves GSDMC and GSDME. Caspase-3 cleaves GSDME to generate GSDMB pores, while caspase-8 directly cleaves GSDMC. The gasdermin N-terminal domains (GSDMD-N, GSDMC-N, GSDME-N) oligomerize to form membrane pores, executing pyroptosis. ASC, apoptosis-associated speck-like protein containing a CARD; ATM, ataxia telangiectasia mutated; Bax, Bcl-2-associated X protein; cIAP1/2, cellular inhibitor of apoptosis protein 1/2; Cyt-C, cytochrome c; DAMPs, damage-associated molecular patterns; FADD, Fas-associated death domain protein; Fodrin, non-erythroid spectrin; GSDMs, gasdermins; IKKα/β, IκB kinase α/β; Lamin A, lamin A/C; LUBAC, linear ubiquitin chain assembly complex; MLKL, mixed lineage kinase domain-like protein; NEMO, NF-κB essential modulator; NLRP3, NOD-like receptor family pyrin domain containing 3; NOXA, NADPH oxidase activator; PARP, poly(ADP-ribose) polymerase; PUMA, p53 upregulated modulator of apoptosis; RIPK1/3, receptor-interacting protein kinase 1/3; ROS, reactive oxygen species; TAB1/2/3, TAK1-binding protein 1/2/3; TAK1, TGF-β-activated kinase 1; TRADD, TNF receptor-associated death domain protein; TRAF2, TNF receptor-associated factor 2.

Other gasdermins besides GSDMD drive secondary pyroptosis, which amplifies inflammatory damage. GSDMD pores release DAMPs, triggering death receptor signaling and activating apoptotic caspases. These caspases then cleave gasdermins to open new pores: caspase-3 cleaves GSDME (57); caspase-8 cleaves GSDMC, GSDMD, and GSDME (58, 59); caspase-6 cleaves GSDMC (60); and caspase-7 cleaves GSDMB (61). This second round of pore formation dumps more DAMPs, fueling the inflammatory response (**Figure 3**).

## 6 Caspases in PANoptosis

**Figure 4:**
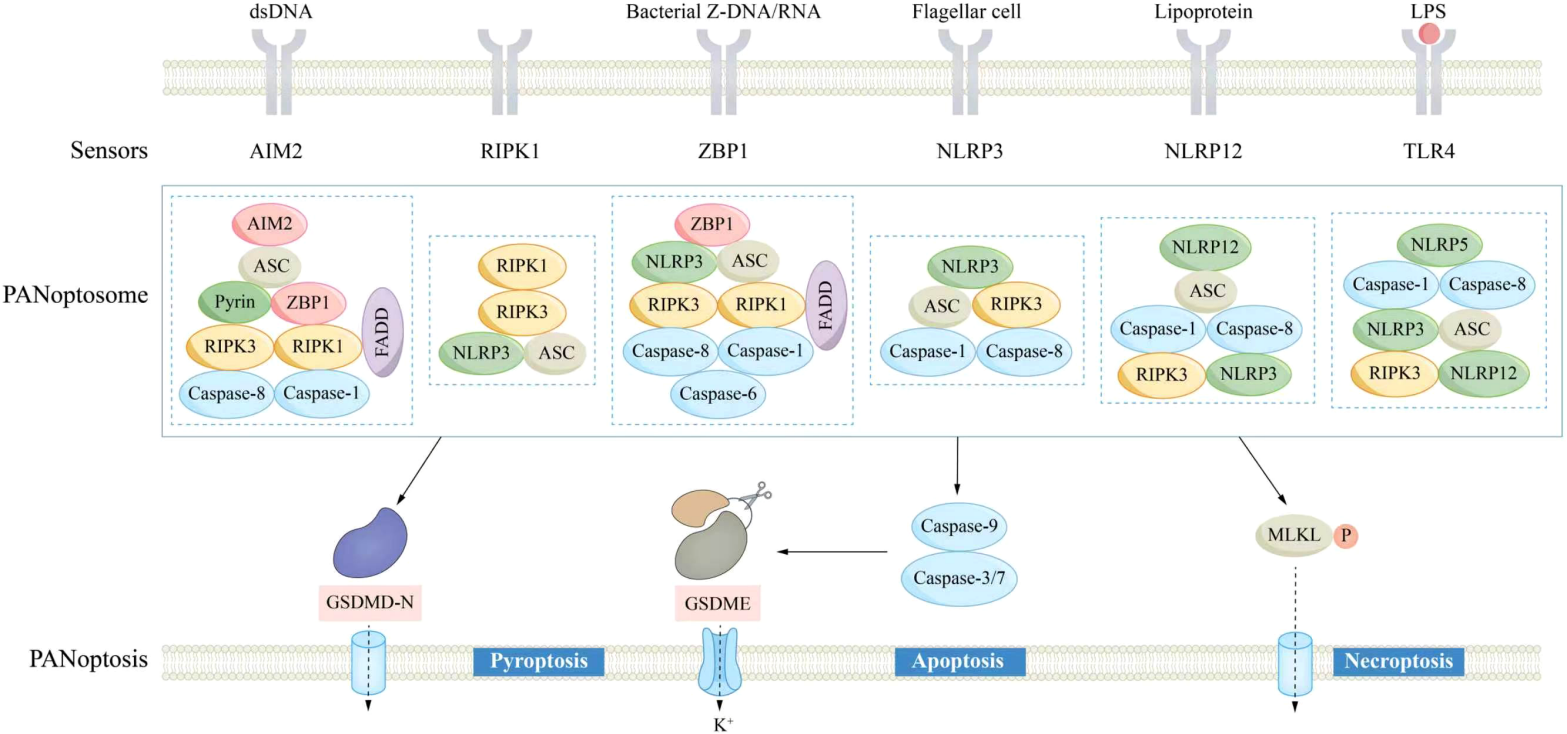
PANoptosome assembly and downstream cell death pathways. Upon sensing distinct PAMPs/DAMPs (dsDNA, bacterial Z-DNA/RNA, flagellin, lipoprotein, and LPS), pattern recognition receptors (AIM2, ZBP1, NLRP3, NLRP12, and TLR4) nucleate the assembly of multimolecular PANoptosomes. These integrated complexes comprise shared and distinct components from pyroptosis, apoptosis, and necroptosis machineries, including RIPK1, RIPK3, FADD, ASC, caspases, and inflammasome sensors (NLRP3, NLRP5, NLRP12, Pyrin). The assembled PANoptosomes trigger distinct cell death outcomes: pyroptosis via GSDMD-N or GSDME pore formation and K⁺ efflux, apoptosis through caspase-9/3/7 activation, and necroptosis via MLKL phosphorylation. AIM2, absent in melanoma 2; ASC, apoptosis-associated speck-like protein containing a CARD; dsDNA, double-stranded DNA; FADD, Fas-associated protein with death domain; GSDMD, gasdermin D; GSDMD-N, gasdermin D N-terminal domain; GSDME, gasdermin E; LPS, lipopolysaccharide; MLKL, mixed lineage kinase domain-like pseudokinase; NLRP3/5/12, NLR family pyrin domain containing 3/5/12; RIPK1/3, receptor-interacting serine/threonine-protein kinase 1/3; TLR4, Toll-like receptor 4; ZBP1, Z-DNA binding protein 1.

PANoptosis is an emerging PCD that differs fundamentally from traditional PCD. Unlike apoptosis, necroptosis, and pyroptosis, these pathways exhibit extensive crosstalk (62, 63). PANoptosis serves as a synergistic defense mechanism by concurrently activating distinct PCD pathways to eliminate infectious threats. However, this protective response is a double-edged sword; aberrant PANoptosome assembly can drive severe immunopathological damage (64, 65). PANoptosis is mediated by the PANoptosome, a macromolecular complex that provides a platform for signal integration (66, 67). Its assembly relies on the coordinated interaction of three molecular tiers: sensors—such as NLRP3, absent in melanoma 2 (AIM2), and Z-DNA binding protein 1 (ZBP1)—which recognize PAMPs or DAMPs, and subsequently recruit adaptor molecules (ASC, FADD) and catalytic effectors (caspase-1/8, RIPK1/3) to initiate the cell death cascade (68). Upon detecting danger signals, it forms a large molecular complex that incorporates core regulators of apoptosis, pyroptosis and necroptosis, facilitating pathway crosstalk and synergistic activation of cell death (69). The deficiency of a single essential component within any individual PCD pathway is insufficient to completely eliminate PANoptosis (**Figure 4**) (70).

Caspase-8 serves as a pivotal regulatory and effector molecule in PANoptosis. On the one hand, activated caspase-8 activates caspase-3 and GSDMD through proteolytic activity, thereby steering cell death toward both apoptosis and pyroptosis (71). On the other hand, inactivated caspase-8 can scaffold RIPK1 assembly, thus redirecting cell death toward necroptosis (72). The compensatory function of caspase-8 ensures that the inhibition of any single PCD pathway cannot completely abrogate PANoptosis (73). In addition, caspase-1 mediates pyroptosis and inflammatory mediator release, and further enhances the assembly of PANoptosomes (74). Furthermore, caspase-6 can also exert a scaffolding effect to facilitate the interaction and assembly of the ZBP1-RIPK3 complex (75).

## 7 Caspase-related PCD modalities in sepsis

Sepsis features a dysregulated immune-inflammatory response to infection, and caspase-mediated PCD modalities play pivotal and context-dependent roles in the initiation, progression, and outcome of sepsis. The dynamic balance of these PCD modalities dictates the severity of immune dysfunction, inflammatory injury, and organ damage, making them critical nodes in sepsis pathogenesis.

As a non-inflammatory PCD modality, apoptosis plays dualistic roles in driving sepsis progression, which are highly dependent on the specific cell subset, activation timing, and stimulation intensity of the apoptotic pathway. In the early stage of infection, apoptosis primarily targets activated immune cells like neutrophils to eliminate pathogens and suppress excessive inflammatory responses, thereby preventing hyperactivation of the immune system and exerting a protective effect against the initial onset of sepsis (76). Neutrophil activation induced by delayed apoptosis associated with caspase-8 inhibition has been reported to contribute to non-specific tissue damage in sepsis (77, 78). However, as sepsis progresses, sustained and excessive apoptosis occurs in the parenchymal cells of vital organs. Current evidence indicates that both intrinsic and extrinsic apoptotic cascades contribute substantially to various sepsis-associated disorders (79, 80). Specifically, elevated levels of caspases and the pro-apoptotic protein Bax, concomitant with decreased anti-apoptotic protein Bcl-2 levels, have been consistently detected in SA-ALI, SA-AKI, SAE, and SIMI (81). Moreover, multiple classic apoptosis-related signaling pathways including phosphatidylinositol 3-kinase (PI3K)-protein kinase B (Akt), mitogen-activated protein kinase (MAPK), p53, and Toll-like receptor 4 (TLR4)- Nuclear factor kappa B (NF-κB) mediate the pathogenesis and progression of these sepsis-associated diseases. For instance, PI3K-Akt pathway activation suppresses Bax and caspase-3 expression, thereby alleviating SIMI (82); conversely, inhibition of the p53, MAPK, and TLR4-NF-κB pathways can downregulate caspase-3 activity to attenuate SA-AKI (83, 84).

Concurrently, the excessive apoptosis of immune cells impairs the clearance of apoptotic bodies. This disrupts tissue homeostasis, exacerbates immune paralysis and organ dysfunction, and ultimately accelerates the disease progression. The excessive death of immune cells is a key driver behind the immunosuppression seen in sepsis (85). Late-stage sepsis features excessive immune cell depletion (NK cells, dendritic cells, macrophages, CD4⁺ T cells, B cells), causing immunosuppression and heightened secondary infection risk (86–88). Meanwhile, extensive apoptosis of immune cells promotes the polarization of these cells toward a tolerogenic phenotype. For example, macrophages switch from a pro-inflammatory to an anti-inflammatory phenotype (89); NK cells show diminished IFN-γ output (90); and dendritic cells show upregulating co-stimulatory molecules CD80, CD86, and CD40 (91). Notably, immunosuppressive regulatory T (Treg) cells are unusually resistant to sepsis-induced apoptosis compared with other immune cells, possibly because their TLR4-NF-κB pathway is inhibited (92). This deficiency markedly increases Treg cell proportion in late infection, thereby further exacerbating immunosuppression (86, 93).

Unlike apoptosis, necroptosis exerts pathogenic effects in sepsis-related injuries primarily by amplifying inflammatory responses and inducing tissue damage. Upon stimulation of the DR pathway, the necroptotic RIPK1-RIPK3-MLKL axis promotes DAMP emission and inflammatory mediator production, consequently stimulating innate immune effector cells and potentiating pro-inflammatory signaling networks (94, 95). Activation of MAPK and NF-κB via necroptosis serves as a major contributor to inflammatory storm and immune cell infiltration (96). TNFR1-mediated necroptotic pathway promotes SA-ALI through NF-κB activation, which subsequently induces the expression of TNF-α, intercellular adhesion molecule 1 (ICAM-1), and IL-6, and facilitates neutrophil infiltration (97). However, in the early stage of infection, necroptosis can also exert protective effects via restricting excessive neutrophil and macrophage activation to attenuate inflammatory responses. *Mlkl⁻/⁻* mice exhibit exacerbated tissue damage, abnormally increased recruitment of immune cells such as neutrophils and macrophages, and excessive production of pro-inflammatory cytokines in sepsis models induced by staphylococcus aureus infection (98).

As the major pathway of necroptosis, RIPK1-RIPK3-MLKL activation is closely associated with sepsis-mediated vital organ injury and represents a potential indicator of sepsis severity (99, 100). Accumulated research reveals that higher RIPK3 and MLKL expression is closely linked to the occurrence of sepsis-related multiple organ dysfunction and patient mortality (101–103). As necroptosis critically regulates the course of sepsis, intervention against the RIPK1-RIPK3-MLKL pathway has become a promising sepsis therapeutic strategy. Necrostatin-1 (Nec-1) is a classic small-molecule inhibitor that exerts its anti-necroptotic effects by specifically inhibiting the phosphorylation of RIPK1, thereby disrupting the downstream signaling cascade (104). During SA-AKI development, Nec-1 intervention inhibits necrosome assembly, lowers the necroptotic rate of renal tubular epithelial cells, and ultimately attenuate SA-AKI (105). Similarly, Nec-1 treatment exerts comparable protective effects in other sepsis-related disorders, thereby mitigating the severity of sepsis (106–109).

Pyroptosis constitutes a crucial regulatory pathway linking inflammatory cascades and immune activation during sepsis pathogenesis. As mentioned, human caspase-4/5 and murine caspase-11 can specifically recognize and bind with high affinity to LPS and its active component lipid A from Gram-negative bacteria through CARD domains (55, 110). This recognition event triggers non-canonical pyroptosis activation, promoting GSDMD cleavage and plasma membrane pore formation. Notably, intracellular K⁺ efflux through GSDMD pores further stimulate NLRP3 inflammasome assembly, which significantly amplifies inflammatory responses through the canonical pyroptosis pathway (56, 111). Dysregulation of this cross-pathway regulatory network results in excessive pro-inflammatory cytokine release, triggering cytokine storms and causing MODS.

Activation of pyroptosis also exerts a dual role during the pathological progression of sepsis. At the initial stage of infection, pyroptosis activation promptly triggers innate immune responses, facilitating the initiation of protective immunity (112). Sepsis patients surviving 7 days post-onset exhibited significantly higher NLRP3 inflammasome activation and caspase-1 expression compared to non-survivors, suggesting that pyroptosis activation during the early infection phase may confer a protective effect by enhancing the host’s innate immune defense against pathogens (113). However, with sepsis progression, sustained activation of pyroptosis triggers excessive inflammatory responses, induces SIRS, and may further lead to septic shock. Studies have shown that most severe injuries associated with sepsis are closely related to aberrant pyroptosis activation. Both septic patients and murine models exhibit pronounced increases in NLRP3, GSDMD, and IL-1β levels (114–116). GSDMD^-/-^ mice exhibit significant resistance to LPS-induced injury (117). The gasdermin D inhibitor Y2 (GI-Y2) attenuates LPS-induced cardiac dysfunction by suppressing pyroptosis (118). Macrophage migration inhibitory factor (MIF) promotes NLRP3-caspase-1-mediated pyroptosis by enhancing p65 phosphorylation, thereby exacerbating SA-AKI (119). Ubiquitination of NLRP3 reduces SA-ALI by suppressing caspase-1 activation and IL-1β maturation (120). Thus, similar to apoptosis and necroptosis, pyroptosis exerts dual effects on sepsis progression, with its functional outcome dependent on the context of activation.

PANoptosis a critical factor contribute to sepsis-associated injury. Dai et al. identified 16 PANoptosis-associated genes that determine sepsis subtypes, and these genes are closely correlated with the expression profiles of B cells, CD16⁺CD14⁺ monocytes and megakaryocyte (121). A bioinformatics analysis identified *CD14, FAS, IL-1β* and *GSDMD* as signature PANoptosis-associated genes correlated with the progression of SA-ALI injury (122). Another study demonstrated that *RIPK2* and *GADD45B* are highly correlated with LPS-induced SIMI, and positively correlated with neutrophils (123). Similar to other PCD modalities, PANoptosis exerts a dual role in the pathological progression of sepsis. PAMPs derived from different sources are specifically recognized by distinct PRRs: for example, ZBP1 recognizes bacterial Z-nucleic acids (Z-DNA/Z-RNA) (124), AIM2 senses double-stranded DNA (dsDNA) (125), NOD-like receptor family members NLRP3 and NLRC4 identify flagellin (126), NLRP12 detects lipoproteins (127), and TLR4 recognizes LPS. The recognition of PAMPs by PRRs facilitates PANoptosome assembly, thereby triggering PANoptosis and eliminating infected cells. However, as sepsis progresses, the processes of pathogen clearance and tissue injury lead to the massive release of endogenous DAMPs, which further amplifies PANoptosis. Overactivated PANoptosis activates inflammasomes and MLKL, resulting in excessive release of TNF-α, IL-1β and IL-6. These cytokines not only directly inflict tissue damage, but also initiate endothelial activation cascades that elevate vascular permeability and drive neutrophil recruitment. TNF-α and IFN-γ synergistically trigger caspase-8-mediated PANoptosis, thereby activating caspase-3, MLKL and GSDMD. This process contributes to multiple organ injury, cytokine storm and depletion of T and B lymphocytes (128). In SA-ALI, ZBP1/NLRP3-mediated PANoptosis exacerbates inflammation and oxidative stress, impairs lung tissue integrity, increases vascular permeability and ultimately induces pulmonary edema (129). In contrast, AIM2-mediated PANoptosis promotes renal tubular epithelial cell death and exacerbates LPS-induced AKI (130).

Targeted therapy against PANoptosome holds great therapeutic potential. For example, Baicalin treatment inhibits mitochondrial DNA release, Z-DNA generation and LPS-induced ZBP1-PANoptosome assembly (131). Similarly, Zn²⁺ inhibits mitochondrial DNA release, which in turn blocks AIM2 activation, attenuates LPS-induced PANoptosis, and potentially mitigate SIMI (132). Additionally, penehyclidine hydrochloride can inhibit the expression of ZBP1 as well as the binding of ZBP1 to FADD and RIPK3, thereby suppressing the inflammatory factor storm and alleviating myocardial injury (133). Targeting downstream effector proteins also yields certain therapeutic efficacy. However, given that PANoptosis simultaneously activates multiple PCD pathways, a combinatorial targeting strategy may exhibit superior efficacy compared with inhibiting a single PCD pathway alone. In the context of SA-ALI, individual blockade of apoptosis/pyroptosis (zVAD-fmk) or necroptosis (Nec-1) failed to reverse cell death. Only the concurrent inhibition of all three pathways significantly improved the viability of pulmonary microvascular endothelial cells and further reduced IL-6 levels (134). In a cecal ligation and puncture (CLP)-induced sepsis model, compared with single-gene knockout or wild-type mice, *RIPK3⁻/⁻GSDMD⁻/⁻* double-knockout mice displayed markedly lower levels of released IL-1β, TNF-α, CXCL2 and CCL3 (135).

Overall, the dual roles of PCD modalities evolve markedly during sepsis. In the early phase, pyroptosis generates antimicrobial IL-1β and IL-18, neutrophil apoptosis clears activated cells and resolves inflammation, and caspase-8 activity suppresses necroptosis (76, 112). This protective configuration breaks down during the transition to established infection. Persistent pathogen load and accumulating DAMPs overwhelm regulatory checkpoints: cFLIP downregulation permits RIPK3-MLKL necroptosis, while caspase-3–mediated GSDME cleavage converts apoptosis into secondary pyroptosis, amplifying rather than resolving inflammation (136). Late sepsis is characterized by lymphocyte depletion and immunosuppression (88), necroptotic organ damage (94), self-amplifying pyroptotic cascades and DAMP-driven PANoptosis triggering cytokine storms (56, 128). The caspase-8/cFLIP ratio and GSDMD-to-GSDME shift serve as critical molecular switches governing these transitions. Such time-dependent kinetics dictate that clinical interventions must be precisely timed (**Table 1**) (113, 120).

**Table 1:** Stage-specific functions and transitions of programmed cell death modalities in sepsis progression.

PCD modality	Early phase: Host defense	Late phase: Pathological injury	Key molecular switch	References
Apoptosis	Neutrophil clearance, inflammation resolution	Lymphocyte depletion, immunosuppression	Bcl-2↓/Bax↑; Fas-FasL↑	(76) (81–84)
Necroptosis	Suppressed by active caspase-8	RIPK3-MLKL activation, DAMP release, organ damage	cFLIP↓; caspase-8 inhibition	(40–43) (94) (98)
Pyroptosis	Antimicrobial IL-1β/IL-18 release, pathogen clearance	Cytokine storm, self-amplifying inflammasome loops	GSDMD→GSDME shift	(56) (57) (136)
PANoptosis	PAMP-driven pathogen elimination	DAMP-driven cytokine storm, multi-organ failure	ZBP1/NLRP3 activation by mitochondrial DNA/HMGB1	(122) (124–130)

↓, downregulated; ↑, upregulated.

## 8 Targeting caspases in sepsis therapy

Caspases sit at the crossroads of cell death and inflammation, making them attractive drug targets in sepsis. However, targeting them requires a delicate balance: suppressing immunopathology without compromising essential host immunity. Pan-caspase inhibitors attempt to arrest sepsis-associated systemic inflammation by targeting multiple caspases simultaneously. Z-VAD and M-920 (targeting caspases-1/3/4/5/6/7/8) have shown promise in CLP models, enhancing survival and preserving lymphocyte populations in primary and secondary lymphoid organs (137). But sweeping blockade of caspase-8 may flipping cells into necroptosis. That liability has pushed the field toward subtype-selective agents, betting that narrower targeting will deliver cleaner, safer outcomes.

Caspase-3, a classic executioner caspase, is activated by upstream caspase-8/9. During sepsis, its overactivation drives apoptosis in parenchymal cells across multiple organs as well as in immune cells such as lymphocytes and macrophages. In pediatric sepsis patients, the apoptosis level of peripheral blood mononuclear cells (PBMCs) was significantly positively correlated with Fas expression (138). Caspase-3 can also promote GSDME-mediated pyroptosis (136). Therefore, targeting caspase-3 stands out as a promising avenue for sepsis therapy. *CASP3* knockout or the selective caspase-3 inhibitor M-725 conferred a survival benefit in CLP-induced septic mice; nevertheless, splenocyte apoptosis persisted at a higher level than that in the sham-operated group, suggesting that caspase-3 inhibition alone can be functionally compensated by other caspases (137). Sevoflurane sedation upregulates Bcl-2, downregulates caspase-3/8/9 expression, and mitigates systemic inflammation and SAE in CLP-induced septic rats (139). A bioinformatics analysis identified S100A9 as a key biomarker in SA-SLI. S100A9 promotes LPS-induced apoptosis of lung epithelial cells via the IL-17-NF-κB-caspase-3 pathway. Overexpression of S100A9 upregulates the transcription of Bax and caspase-3, whereas knockout of S100A9 reverses these effects (140). Clemastine pretreatment exerts a marked protective effect against CLP-induced SA-AKI in rats by regulating α-Klotho-TLR4-NF-κB-caspase-3 pathway-mediated apoptosis (141). Hepatic Caspase-3 and GSDME expression was profoundly augmented following CLP-induced liver injury. Blocking Caspase-3 or GSDME lessened liver pathology and decreased IL-1β and TNF-α levels (142).

Given the core driving role of pyroptosis in the initiation and progression of the inflammatory storm in sepsis, targeting caspases involved in the pyroptosis pathway is also expected to become an important therapeutic strategy for sepsis. In the canonical pyroptosis pathway, targeting each step of inflammasome assembly can effectively regulate the maturation and activation of caspase-1. In CLP-induced SA-AKI, the levels of NLRP3, ASC, and caspase-1 were significantly elevated. Overexpression of Sirtuin 3 (Sirt3) reduces ROS and disrupts the NLRP3-caspase-1 axis in renal tubular epithelial cells, thereby preventing pyroptotic demise and attenuating IL-1β/IL-18 discharge (143). Under physiological or oxidative stress concentrations, 4-hydroxynonenal (HNE) directly engages NLRP3 through cysteine-mediated binding, thereby obstructing mammalian NIMA-related kinase 7 (NEK7) complex formation and impeding inflammasome construction and functional priming (144). This dampens caspase-1/GSDMD processing, thereby halting macrophage pyroptosis and blunting IL-1β/IL-18 release (145). In contrast, dihydrotanshinone I (DHT) exerts no effect on ROS production or NLRP3-NEK7 binding. Instead, it inhibits NLRP3-induced ASC oligomerization, prevents inflammasome assembly, and thereby suppresses caspase-1 activation and septic shock (146).

The non-canonical caspase-4/5/11 axis is also integral to sepsis pathophysiology. Radiation-induced DNA damage leads to the release of cytoplasmic DNA, thereby engaging cyclic GMP-AMP synthase (cGAS) and instigating type I interferon signaling. This activates caspase-11, driving pyroptosis of pulmonary endothelial cells and aggravating SA-ALI. Inhibition of cGAS or caspase-11 can help mitigate the aforementioned symptoms (147). Silymarin can dose-dependently inhibit the auto-cleavage of pro-caspase-11, block caspase-11-mediated pyroptosis and inflammatory mediator secretion, and rescue mice from LPS-induced acute lethal sepsis (148). Phillyrin upregulates miR-203a to inhibit the expression of caspase-4 (human), caspase-11 (mouse) and caspase-B (zebrafish), thereby alleviating the inflammatory response and multiple organ injury associated with sepsis (149). MiR-203a directly interacts with the *CASP4* 3’UTR and suppresses its post-transcriptional expression.

Furthermore, since cytosolic LPS instigates the non-canonical pyroptosis cascade, blocking its internalization can also effectively inhibit caspase-4/5/11-mediated pyroptosis. The principal LPS internalization routes include clathrin-mediated outer membrane vesicles (OMVs) endocytosis (150), receptor for advanced glycation end products (RAGE)-mediated endocytosis of high-mobility group box 1 (HMGB1)-LPS complexes (151), and scavenger receptor-mediated endocytosis of LPS-LPS binding protein (LBP) complexes (152, 153). HMGB1 released by hepatocytes may contribute to sepsis lethality via caspase-11-dependent signaling. HMGB1 binds to LPS and undergoes RAGE-mediated endocytosis into the lysosomes within macrophages and endothelial cells. Subsequently, HMGB1 induces lysosomal rupture, which leads to the release of LPS into the cytoplasm and subsequent caspase-11 activation, thereby exacerbating the systemic inflammation (151). Specific knockout of HMGB1 or RAGE in hepatocytes effectively reduces inflammatory cytokine secretion and improves survival in mice with CLP-induced sepsis. Another protein closely associated with non-canonical pyroptosis is galectin-3 (Gal3). Gal3 enhances free LPS endocytosis by binding to RAGE. It also interacts with CD44 to orchestrate the clathrin-independent endocytosis of LPS-containing bacterial OMVs into cells. Gal3 overexpression exacerbates caspase-4/11-mediated pyroptosis and SA-AKI, and its inhibition confers protection against renal tubular epithelial cell injury (154). Dynein Light Chain LC8-Type 2 (DYNLL2) is upregulated in sepsis and predicts poor prognosis. The DYNLL2-p21-activated kinase 1 (PAK1) axis mediates cytoplasmic LPS release and caspase-11-dependent pyroptosis by regulating the endocytosis of OMVs derived from Gram-negative bacteria (155). DYNLL2 knockdown significantly inhibits Escherichia coli-induced macrophage pyroptosis.

Caspase-7 also emerges as a promising therapeutic target in sepsis. A study demonstrated that *CASP7*⁻/⁻ mice, but not *CASP3*⁻/⁻ mice, exhibited a significant reduction in LPS-induced lymphocyte apoptosis (156). Interestingly, caspase-7 cuts GSDMB at the D91 site, which is a crucial mechanism for inhibiting non-canonical pyroptosis (61). GSDMB mediates pyroptosis through two distinct pathways: first, granzyme A secreted by lymphocytes truncates GSDMB at K244 to induce non-cell-autonomous pyroptosis, Second, full-length GSDMB recruits caspase-4 and enhances its proteolytic efficiency, thereby promoting cell-autonomous non-canonical pyroptosis (48, 157). Caspase-7 activation triggers GSDMB truncation at the D91 site and thus suppresses non-canonical pyroptosis, functioning as an essential self-protective strategy for the host to prevent excessive inflammatory activation during infection. In THP-1 cells infected with Escherichia coli or Salmonella Typhimurium, caspase-7 knockdown results in decreased cleavage of GSDMB, enhanced GSDMD cleavage, and elevated IL-1β secretion, and a marked increase in pyroptotic cells, whereas knockdown of caspase-3, -6, or -8 shows no such effects (61). Therefore, the advantages and disadvantages of targeting caspase-7 should be fully taken into account in sepsis therapy.

Caspase-8 is a pivotal regulatory node within the PANoptosome, coordinating upstream signal integration and governing the selection of downstream PCD modalities based on the initiating stimulus and cellular milieu. It initiates apoptosis via caspase-3, triggers pyroptosis through direct or indirect cleavage of GSDMD, and modulates necroptosis by regulating RIPK1 (40, 158). Consistent with its pivotal regulatory function in PCD and inflammation, a study reported that caspase-8 was significantly associated with sepsis mortality, and non-survivors displayed considerably elevated serum caspase-8 levels relative to survivors (159). These findings point to caspase-8 as a key player in sepsis. Caspase-8-associated PANoptosis is central to sepsis pathogenesis. For instance, TNF-α-instigated DR pathway priming and caspase-8-mediated apoptosis contribute to the exacerbation of SA-AKI (160). The selective caspase-8 antagonist IETD-fmk can suppress LPS-induced monocyte activation and redirects them to necroptosis, thereby alleviating SIRS (161). TNF-α/IFN-γ potently induces SARS-CoV-2-associated PANoptosis via the JAK-STAT1-IRF1 signaling pathway, fueling inducible nitric oxide synthase (iNOS) induction and abundant NO production. The ensuing NO further triggers the FADD-caspase-8 cascade, leading to GSDME processing, caspase-3/7 maturation, and MLKL activation, thereby completing PANoptosis (128). Dual blockade of TNF-α/IFN-γ, or targeted intervention against their downstream critical signaling nodes, effectively mitigates pathological damage associated with cytokine storm (162, 163). Given the indispensable contribution of caspase-8 to PANoptosis execution and sepsis pathogenesis, targeted regulation of its activity has become a focus of sepsis intervention research.

As mentioned earlier, cFLIP forms a heterodimer with caspase-8, which inhibits the enzymatic activity of caspase-8 while preserving its scaffolding function, thereby promoting cell survival (41–43). In CLP-induced sepsis, cFLIP expression is significantly downregulated in essential organs including the brain, lung, liver, and kidney, and could not suppress endothelial apoptosis (164). In a murine model of Fas-associated lethal multi-organ failure, intraperitoneal administration of TAT-cFLIP markedly suppresses caspase-8 and caspase-3 activation, thus prolonging survival (165). Single knockout of either caspase-8 or cFLIP results in TNF-related tissue damage and inflammation, but with distinct dependencies on RIPK3. TNF neutralization or simultaneous knockout of both caspase-8 and cFLIP significantly attenuates tissue damage and sepsis (166). Xia et al. identified Nemo-like kinase (NLK) as an upstream regulator of caspase-8. NLK overexpression in sepsis facilitates its recruitment to the FADD-caspase-8-RIPK1 PANoptosome. By binding to the DED domain of caspase-8, NLK promotes its recruitment and proximal activation, thereby promoting pyroptosis and apoptosis in macrophages while inhibiting RIPK1/3-mediated necroptosis. Depletion of NLK impaired caspase-8 activation, suppressed pyroptosis and apoptosis during PANoptosis, and shifted the cell death mode toward necroptosis, thereby mitigating sepsis-driven hyperinflammation and multi-organ damage while enhancing murine survival outcomes (167). In another study, silencing caspase-8 failed to significantly inhibit lung epithelial cell death and inflammatory responses in ALI mice, in contrast to silencing Fas. This may be because inhibition of Fas blocks the entire DR pathway, whereas Fas-related non-apoptotic inflammatory signals are independent of caspase-8 but mediated by cFLIP (42). Furthermore, since the DISC complex remains intact, inhibition of caspase-8 can also trigger necroptosis, thereby aggravating inflammatory responses and neutrophil infiltration (168). This illustrates a broader issue with targeted inhibition in sepsis: the various cell death pathways are extensively interconnected, and blocking one frequently just reroutes the signal to another. In PANoptosis, the integrated architecture of the PANoptosome means that disabling any single component is insufficient to abort cell death, as remaining constituents maintain pathway activity (70). Consequently, concurrent blockade of multiple nodes, as demonstrated by the superior efficacy of combined necroptosis and pyroptosis inhibition in RIPK3⁻/⁻GSDMD⁻/⁻ mice, may be required to overcome this mechanistic redundancy and achieve meaningful therapeutic benefit (135). Efferocytosis, the clearance of apoptotic cells by macrophages, is typically an immunologically silent and anti-inflammatory process that promotes resolution of inflammation. However, Muendlein et al. reported that TNF converts macrophage efferocytosis of dying neutrophils into caspase-8-dependent pyroptosis during sepsis/SIRS, mediating IL-1β maturation and serve as a major contributing factor to septic tissue injury. TNF combined with dying neutrophil efferocytosis suppresses the TGFβ-activated kinase 1 (TAK1)/NF-κB pathway, reduces cFLIP expression, and promotes the assembly of the ZBP1-caspase-8-RIPK1 complex, thereby mediating caspase-8/GSDMD activation and triggering pyroptosis. Meanwhile, efferocytosis upregulates pro-IL-1β transcription by activating the PLCγ/MAPK pathway downstream of phosphatidylserine (PS) receptors, and achieves IL-1β maturation and release through GSDMD-mediated cleavage (169). Consequently, dual inhibition of TNF and PS pathways may serve as a promising therapeutic strategy for sepsis. Nevertheless, careful consideration is required due to the organ-dependent functions of efferocytosis during sepsis: suppressing T cell immunoglobulin mucin 3 (TIM3) diminishes splenic macrophage-mediated clearance of neutrophils in murine models, yet aggravates cellular injury in pulmonary and renal tissues. Furthermore, Jiang et al. reported that integrin α2β (ITGA2B) in platelets is protectively upregulated during sepsis. ITGA2B upregulates protein tyrosine phosphatase non-receptor type 6 (PTPN6) in megakaryocytes via the transcription factors Nfkb1/Rel, thereby simultaneously inhibiting caspase-8 and MLKL to suppress platelet apoptosis and necroptosis (170). Deficiency of ITGA2B results in massive apoptosis and necroptosis of platelets, leading to thrombocytopenia and impaired vascular integrity; these phenotypes can be reversed by transfusion of wild-type platelets.

**Figure 5:**
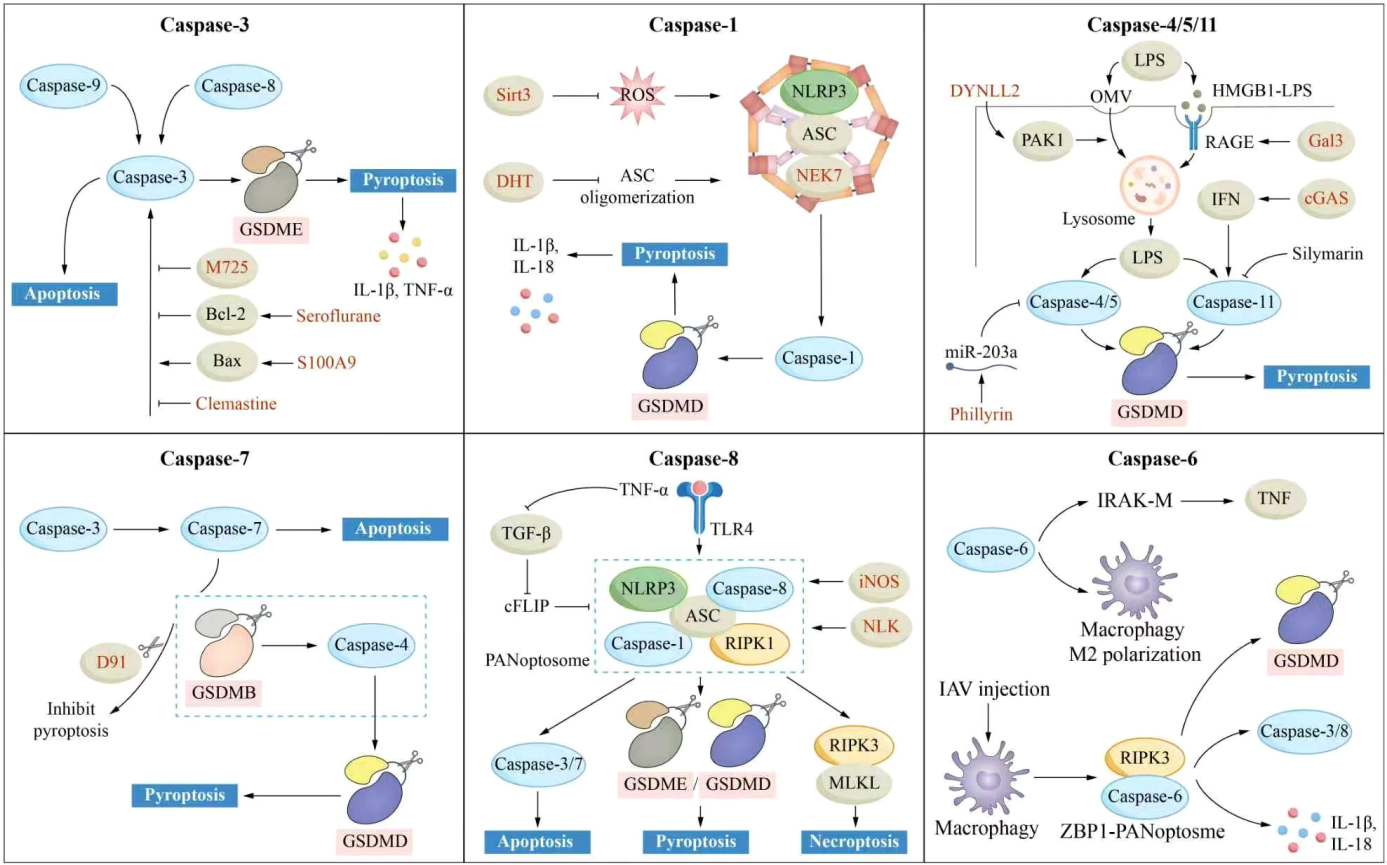
Targeting different caspases for the modulation of apoptosis, pyroptosis, necroptosis, and PANoptosis in sepsis. Caspase-3: targeted by M-725, Bcl-2 upregulation (seroflurane), Bax inhibition (S100A9 knockout), and clemastine; mediates apoptosis and GSDME-dependent pyroptosis. Caspase-1: regulated by Sirt3 (ROS reduction), DHT (ASC oligomerization blockade), and NLRP3-NEK7 disruption; drives canonical pyroptosis via GSDMD cleavage. Caspase-4/5/11: inhibited by miR-203a (phillyrin), silymarin, and DYNLL2/PAK1 blockade; activated by cytosolic LPS (via OMV, HMGB1-LPS/RAGE, or cGAS-IFN pathways) to execute non-canonical pyroptosis. Caspase-7: cleaves GSDMB at D91 to suppress non-canonical pyroptosis; activated downstream of caspase-3. Caspase-8: central PANoptosome regulator; modulated by cFLIP, NLK, and TNF-α/TLR4 signaling; directs apoptosis (caspase-3/7), pyroptosis (GSDME/GSDMD), or necroptosis (RIPK3/MLKL). Caspase-6: cleaves IRAK-M to promote TNF production and M2 macrophage polarization; scaffolds ZBP1-RIPK3 complex during IAV infection to drive PANoptosome assembly and IL-1β/IL-18 release. ASC, apoptosis-associated speck-like protein containing a CARD; cFLIP, cellular FLICE-like inhibitory protein; cGAS, cyclic GMP-AMP synthase; DHT, dihydrotestosterone; DYNLL2, Dynein Light Chain LC8-Type 2; Gal3, galectin-3; GSDM, gasdermin; HMGB1, high mobility group box 1; IAV, influenza A virus; IFN, interferon; IL, interleukin; IRAK-M, interleukin-1 receptor-associated kinase M; iNOS, inducible nitric oxide synthase; LPS, lipopolysaccharide; MLKL, mixed lineage kinase domain-like protein; NEK7, NIMA-related kinase 7; NLK, Nemo-like kinase; NLRP3, NOD-like receptor family pyrin domain containing 3; OMV, outer membrane vesicle; PAK1, p21-activated kinase 1; PARP, poly(ADP-ribose) polymerase; RAGE, receptor for advanced glycation end products; RIPK1/3, receptor-interacting protein kinase 1/3; ROS, reactive oxygen species; Sirt3, sirtuin 3; TGF-β, transforming growth factor-beta; TLR4, Toll-like receptor 4; TNF-α, tumor necrosis factor-alpha; ZBP1, Z-DNA binding protein 1.

Caspase-6 was traditionally recognized as an apoptotic executioner. However, recent studies suggest this view is too narrow (171–173). For instance, direct contact with neutrophils can trigger TLR2/4-independent autoactivation of caspase-6 in macrophages (174). Caspase-6 then directly cleaves IL-1 receptor-associated kinase-M (IRAK-M) at the VTVD¹³⁵ site, thereby mediating TNF production. In IL-4-induced anti-inflammatory macrophages, caspase-6 is markedly upregulated without triggering apoptosis, thereby promoting anti-inflammatory polarization (175). Inhibition of caspase-6 significantly reduces the abundance of characteristic anti-inflammatory hallmarks, exemplified by IL-10, MMP-2, and MMP-9. In addition, during influenza A virus (IAV) infection, caspase-6 scaffolds RIPK3 to enhance ZBP1-RIPK3 interaction and ZBP1-PANoptosome assembly (75). In *Casp6⁻/⁻* macrophages, IAV infection results in global reductions in caspase-1/-3/-8 activation, IL-1β/IL-18 secretion, GSDMD cleavage, and MLKL phosphorylation, accompanied by markedly reduced cell death (176). However, although current studies have established that caspase-6 plays pivotal roles in innate immunity, ZBP1-NLRP3 inflammasome activation, and PANoptosis, its therapeutic potential as a target in sepsis remains to be further validated through additional investigations (**Figure 5**) (**Table 2**).

**Table 2:** Caspase-targeted regulators in sepsis therapy.

Regulator	Target caspase	Mechanism	References
Z-VAD	Caspase-1/3/4/5/6/7/8	Pan-caspase inhibitor; blocks excessive PCD and inflammatory cascades	(137)
M-920	Caspase-1/3/4/5/6/7/8	Pan-caspase inhibitor; improves survival in CLP-induced septic mice and reduces mature T/B lymphocyte apoptosis in thymus and spleen	(137)
M-725	Caspase-3	Inhibits apoptosis; confers survival benefit in CLP-induced septic mice	(137)
Sevoflurane	Caspase-3/8/9	Upregulates Bcl-2, downregulates caspase-3/8/9 expression, mitigates systemic inflammation and SAE	(139)
Clemastine	Caspase-3	Regulates α-Klotho-TLR4-NF-κB-caspase-3 pathway-mediated apoptosis	(141)
Sirtuin 3	Caspase-1	Reduces ROS and disrupts NLRP3-caspase-1 axis in renal tubular epithelial cells, preventing pyroptotic demise	(143)
4-hydroxynonenal	Caspase-1	Directly engages NLRP3 through cysteine-mediated binding, obstructing NEK7 complex formation and impeding inflammasome construction; dampens caspase-1/GSDMD processing	(144) (145)
Dihydrotanshinone I	Caspase-1	Inhibits NLRP3-induced ASC oligomerization, prevents inflammasome assembly	(146)
Silymarin	Caspase-11	Dose-dependently inhibits auto-cleavage of pro-caspase-11	(148)
Phillyrin	Caspase-4/11	Upregulates miR-203a to inhibit expression of caspase-4 (human), caspase-11 (mouse) and caspase-B (zebrafish)	(149)
IETD-fmk	Caspase-8	Suppresses LPS-induced monocyte activation and redirects them to necroptosis, alleviating SIRS	(161)
TAT-cFLIP	Caspase-8/3	Markedly suppresses caspase-8 and caspase-3 activation in Fas-associated lethal multi-organ failure model	(165)
ITGA2B	Caspase-8	Upregulates PTPN6 in megakaryocytes via the transcription factors Nfkb1/Rel, thereby simultaneously inhibiting caspase-8 and MLKL	(170)
S100A9	Caspase-3	Promotes apoptosis via the IL-17-NF-κB-caspase-3 pathway; upregulates transcription of Bax and caspase-3	(140)
Galectin-3	Caspase-4/11	Binds to RAGE to enhance free LPS endocytosis and interacts with CD44 to orchestrate clathrin-independent endocytosis of LPS-containing bacterial OMVs	(154)
DYNLL2	Caspase-11	DYNLL2-PAK1 axis mediates endocytosis of Gram-negative bacterial OMVs and cytoplasmic LPS release	(155)
NLK	Caspase-8	Binds to the DED domain of caspase-8, promotes its recruitment and proximal activation, facilitates recruitment to the FADD-caspase-8-RIPK1 PANoptosome	(169)

While the studies reviewed above provide mechanistic insights into caspase-mediated PCD in sepsis, translating preclinical caspase-PCD research to human sepsis faces significant biological and clinical barriers. Mice need much higher LPS doses than humans because they handle endotoxin better. The result is a sharp, short-lived inflammatory spike that doesn’t match the drawn-out immune dysfunction seen in human sepsis. There are also real species differences: mice use caspase-11 for non-canonical pyroptosis, while humans use caspase-4/5. And in the clinic, sepsis patients almost always have other conditions and are on drugs that affect immunity—something simple mouse models usually miss (177).

## 9 Conclusion

Caspases are conserved cysteine proteases that sit at the center of sepsis pathology by controlling programmed cell death (178). Normally, caspase-driven PCD is a key defense against infection. But when this system spins out of control, it triggers immune exhaustion, cytokine storms, and vascular collapse. Although caspase inhibitors have shown survival benefits in animal models, these findings have so far failed to translate into clinical efficacy.

PANoptosome composition is still poorly understood. ZBP1, AIM2, and NLRP3 recognize specific pathogens, but which sensor dominates during polymicrobial sepsis remains unclear (124–127, 179). How the stoichiometry of these components affects inflammation and cell death also needs clarification. This matters because it may explain why sepsis produces different clinical outcomes—ARDS in some patients, acute kidney injury in others.

Another blind spot is the timing and location of caspase activation. Traditional methods cannot capture the temporal hierarchy, making it unclear if caspase-8 activation at the DISC is a prerequisite for inflammasome assembly. It’s also unclear how cell death moves between cells—via DAMPs, direct contact, or other routes. Real-time biosensors could answer this, helping us design treatments that stop the harmful spread while keeping protective immunity intact.

One major problem is that caspases behave very differently depending on the situation. The same caspase-8 can trigger lymphocyte death and immunosuppression, or it can compromise the endothelial barrier, depending on the cell type and environment (159, 160). Macrophage pyroptosis is mostly inflammatory, yet neutrophil pyroptosis may be protective against bacteria (47, 112). Single-cell data is starting to reveal which cells take the biggest hit, but we haven’t yet connected these specific caspase events to clinical outcomes (121).

Selective inhibition poses a particularly thorny problem. Broad caspase blockade wipes out both harmful and protective PCD, weakening host defense (137). More targeted strategies carry their own complications: blocking caspase-7 spares lymphocytes but unleashes GSDMB-driven non-canonical pyroptosis (61, 156). Blocking caspase-8 can shunt cells toward necroptosis, worsening inflammation rather than preventing it (40, 161). We need drugs that can tell pathological from normal activation apart—perhaps by detecting disease-specific post-translational modifications or activation confined to particular cellular compartments.

Human systems immunology offers a way forward. Single-cell RNA sequencing has already identified sepsis endotypes with distinct cell death signatures (121, 122), and spatial transcriptomics can now map where different death modalities occur within injured organs. Whether apoptotic, pyroptotic and necroptotic zones overlap or remain segregated is now an addressable question. Combining these approaches with proteomic, metabolomic and epigenomic data may reveal upstream regulatory nodes that are more druggable than caspases themselves. Longitudinal profiling of patient specimens, rather than reliance on murine LPS or CLP models, will be essential for stratifying patients by their dominant death mechanisms and matching them to appropriate therapies.

In conclusion, caspases play a pivotal role in orchestrating PCD and inflammation during sepsis. Comprehensive elucidation of caspase mediated PCD mechanisms promises to both deepen pathogenetic understanding and catalyze the creation of precision treatments, thereby ameliorating clinical outcomes and quality of survival in individuals afflicted with sepsis and septic shock.
